# Supplementary material for: Getting More Out of Biomedical Documents with GATE's Full Lifecycle Open Source Text Analytics
Source: PLoS Comput Biol. 2013 Feb 7;9(2):e1002854. doi: 10.1371/journal.pcbi.1002854 (PMC3567135; doi:10.1371/journal.pcbi.1002854)
Supplement: Dataset S1 — GATE software. Dataset S1 bundled with this paper contains a distribution of GATE (or see http://gate.ac.uk/download/). (TGZ) [file pcbi.1002854.s001.tgz › plos-gate/src/gate/resources/gate.ac.uk/tests/ft-bt-03-aug-2001.html]

Guardian Unlimited | Archive Search


|  |  |
| --- | --- |
|  |  |


|  |  |
| --- | --- |
|  | **Go to:**  Guardian Unlimited home Politics Books EducationGuardian.co.uk Clearing Film Sport Football Jobs MediaGuardian.co.uk SocietyGuardian.co.uk Money Shopping Travel The Observer Work Search the archive ---------------------- News Audio Net news Special reports The Guardian UK latest The weblog The newslist The wrap World latest Arts latest ---------------------- Crossword Content distribution Events / offers Help / contacts Information Life online Notes & Queries Style guide Travel offers TV listings Weather Web guides ---------------------- Guardian Weekly Money Observer |


|  |  |  |  |  |  |  |  |  |  |  |  |  |  |  |  |  |  |  |  |  |  |  |  |  |  |  |  |  |  |  |  |  |  |  |  |  |  |  |
| --- | --- | --- | --- | --- | --- | --- | --- | --- | --- | --- | --- | --- | --- | --- | --- | --- | --- | --- | --- | --- | --- | --- | --- | --- | --- | --- | --- | --- | --- | --- | --- | --- | --- | --- | --- | --- | --- | --- |
| Network home |  |  |  |  | UK news |  |  |  |  | World latest |  |  |  |  | Books |  |  |  |  | Money |  |  |  |  | Film |  |  |  |  | Society |  |  |  |  | The Observer |  |  |  |
|  | | | | | | | | | | | | | | | | | | | | | | | | | | | | | | | | | | | | | | |
| Politics |  |  |  |  | Education |  |  |  |  | Shopping |  |  |  |  | Work |  |  |  |  | Football |  |  |  |  | Jobs |  |  |  |  | Media |  |  |  |  | Search |  |  |  |


|  |  |  |
| --- | --- | --- |
|  |  |  |
|  |  | **Earth Lease steps up BT campaign**   **Richard Wray**  **Guardian** **Friday August 3, 2001** Pressure was mounting on British Telecom last night to reopen negotiations with Earth Lease after the investment consortium met telecoms regulator Oftel to outline its plan to buy BT's network of local phone lines. Earth Lease is also understood to have called for a meeting with government officials within the next 10 days to explain how its sale and leaseback plan could be used to speed up the introduction of broadband services. The consortium, headed by financiers Babcock & Brown, is attempting to build credibility with officials as a way of forcing BT to reconsider its initial dismissal of an £8bn bid for the country's 28m local copper lines. It has already received indications of support from some of BT's largest customers who use the "local loop" to provide services such as video-on-demand. Earth Lease is expected to call a further meeting with Oftel later this month. However, the consortium has ruled out a direct appeal to shareholders, believing that for any bid to work it must have the cooperation of BT's management and engineers. To bring this about, Earth Lease is willing to discuss the conditions that BT would need to place on any deal handing over control of the local network. Such conditions would include the ability of BT engineers to enter prem ises in order to upgrade equipment. The Earth Lease bid comes at a time when BT is lobbying Oftel to end the controls which have capped the price it can levy in the UK market since privatisation in the 1980s. The local network plays a crucial role in the regulator's review. Spinning off BT's local network would make the regulator's job easier, as it would bring about the effective split of BT's wholesale operation from its retail arm, which could then be treated just as any other telecom provider. BT has been the subject of intense criticism from rival operators over the past year for its handling of the introduction of competition into the local loop. Yesterday the European commission stepped up its drive for competition in the local loop - a process that was supposed to have started throughout the region at the beginning of last month. The commission is asking new entrants to the telecoms market to provide any information about incumbent operators who limit access to local copper networks as it tries to revitalise competition. The commission intends to report on the state of the industry by the end of the year and will take action against any operators it believes are guilty of anti-competitive behaviour. Analysts said that it appears to be willing to take a more rigorous approach to telecoms competition. |
|  |  |  |


|  |  |  |  |  |
| --- | --- | --- | --- | --- |
|  | Guardian Unlimited © Guardian Newspapers Limited 2001 | | | |
